# Randomised controlled study in the primary healthcare sector to investigate the effectiveness and safety of auriculotherapy for the treatment of uncomplicated chronic rachialgia: a study protocol

**DOI:** 10.1186/1472-6882-8-36

**Published:** 2008-07-06

**Authors:** Jorge Vas, Inmaculada Aguilar, M Ángeles Campos, Camila Méndez, Emilio Perea-Milla, Manuela Modesto, Paloma Caro, Francisco Martos, Antonio J García-Ruiz

**Affiliations:** 1 Pain Treatment Unit, Primary Healthcare Centre, Dos Hermanas, Spain; 2 Andalusian Public Health System, Sevilla, Spain; 3 Support Research Unit (Public Health and Epidemiology Network Biomedical Research Centre. CIBERESP), Costa del Sol Hospital, Marbella, Spain; 4 Doña Mercedes Primary Healthcare Centre, Dos Hermanas, Spain; 5 Department of Pharmacology, Malaga University, Spain

## Abstract

**Background:**

Uncomplicated chronic rachialgia is a highly prevalent complaint, and one for which therapeutic results are contradictory. The aim of the present study is to evaluate the effectiveness and safety of treatment with auriculopressure, in the primary healthcare sector, carried out by trained healthcare professionals via a 30-hour course.

**Methods/Design:**

The design consists of a multi-centre randomized controlled trial, with placebo, with two parallel groups, and including an economic evaluation. Patients with chronic uncomplicated rachialgia, whose GP is considering referral for auriculopressure sensory stimulation, are eligible for inclusion. Sampling will be by consecutive selection, and randomised allocation to one of the two study arms will be determined using a centralised method, following a 1:1 plan (true auriculopressure; placebo auriculopressure). The implants (true and placebo) will be replaced once weekly, and the treatment will have a duration of 8 weeks. The primary outcome measure will be the change in pain intensity, measured on a visual analogue scale (VAS) of 100 mm, at 9 weeks after beginning the treatment. A follow up study will be performed at 6 months after beginning treatment. An assessment will also be made of the changes measured in the Spanish version of the McGill Pain Questionnaire, of the changes in the Lattinen test, and of the changes in quality of life (SF-12). Also planned is an analysis of cost-effectiveness and also, if necessary, a cost-benefit analysis.

**Discussion:**

This study will contribute to developing evidence on the use of auriculotherapy using Semen vaccariae [wang bu liu xing] for the treatment of uncomplicated chronic rachialgia.

**Trial registration:**

Current Controlled Trials ISRCTN01897462.

## Background

Uncomplicated chronic rachialgia (UCR) is characterised by pain in the vertebral and paravertebral areas, the intensity of which varies depending on postures, movements and effort. It is usually associated with a painful limitation of mobility of the spine and sometimes with extensive or irradiated pain. The diagnosis of such complaints excludes fractures, direct traumas and systemic disease, but includes structural alterations of the spine, such as disc hernias or facetary alterations [1]. However, only 5% of the mechanical disorders of the spinal column that are treated in primary healthcare result from a structural alteration of the spine. The remaining 95% are comprised of non-specific neck and back pains [2]. Of all these locations, the lumbar region is most prevalent; between 60 and 70% of adults are estimated to have suffered this complaint at some time. Moreover, it tends to be persistent or recurrent [3] and in 95% of cases it is of non-specific muscular-skeletal origin [4]. In Spain, work time lost due to back pain is an average of 22 days per year, with an average cost of 1,260 euros per worker; the complaint represents 19–25% of the total expenditure on benefits for temporary sickness pay, and the total effect of back pain on the workplace has been estimated at an average of 55,388 days lost every year [5]. 90% of the patients with non-specific back pain return to work within six week [6]. Neck pain, on the other hand, is a problem that has been estimated to effect, at some time in life, 30% of men and 43% of women [7], becoming chronic among 10% of men and 17% of women. It is currently the second most important rheumatic cause for occupational invalidity [8]. Although the aetiology of neck pain is varied, ranging from tumoral problems to traumatisms, infections, inflammatory diseases and congenital diseases, in most cases no systemic causes can be identified, and so the largest such group is labelled as "non-specific neck pain". A recent study carried out in Finland estimated the annual prevalence of back pain to be 17% [9].

The treatment for non-specific back pain is usually conservative, and the standard procedure is to recommend pharmacological treatment with analgesic medication such as non-opiate analgesics, non steroidal anti-inflammatory drugs (NSAIDs), myorelaxants and opiates. The evidence derived from 51 studies examined in a systematic review [10] suggests that NSAIDs are effective for short-term symptomatic relief for patients with back pain, but there does not seem to be a specific variety of NSAID that is clearly more effective than any other. Myorelaxants are effective in treating non-specific back pain, but their adverse side effects mean they must be used with caution. Trials are needed to determine whether myorelaxants are more effective than analgesics or NSAIDs [11]. Multidisciplinary biopyschosocial rehabilitation for sub-acute pain in adults of working age has been analysed in a systematic review [12], which concluded that there exists a moderate degree of proof of the positive effectiveness of multidisciplinary rehabilitation. As this evidence is based on studies that presented some kind of methodological defect, and as various types of expensive multidisciplinary rehabilitation are commonly employed, there is an obvious need for high-quality trials to be carried out in this field [13].

Acupuncture and related techniques such as auriculotherapy have been used as therapeutic methods in China for over 2000 years, and are gaining increasing acceptance in the West, where their use has increased considerably in recent decades, especially for pathologies characterised by the presence of pain [14]. Stimulation of the auricular pavilion is usually employed as a technique associated with somatic acupuncture for alleviating pain, and also for sleep disorders, anxiety [15,16] or complaints of the autonomic nervous system [17], but it may also be used alone for the treatment of diverse muscular-skeletal pathologies [18-23], probably taking effect by means of an endorphinergic mechanism [24]. A wide variety of mappings have been made of the auricular pavilion, based on a supposed interconnection between the ear and the rest of the body, such that muscular-skeletal structures would be reflected, somatotopically, in the auricular pavilion [25,26]. Thus, any complaint of the muscles or skeleton would have an accurate representation within the ear, made evident as an increase in sensitivity to pressure or as a decrease in the electrical resistance of the skin [27]. The treatment usually consists of locating the sensitive points and then stimulating them with needles, which may be left in place for several days, or by the implantation of vaccaria seeds (*wang bu liu xing*), held within the auricular pavilion by means of small pieces of surgical tape. Nevertheless, as yet no clear relation has been determined between the somatic locations where muscular-skeletal pain is felt and the sensitive points within the auricular pavilion [28].

We have designed this randomised controlled multicentre study to investigate the effectiveness and safety of sensory stimulation via auricular implants in patients with UCR, treated by healthcare professionals (15 doctors and nurses) at 10 healthcare clinics in the Sevilla-Sur Healthcare District (within the Andalusian Public Health System). The primary specific goal is to assess effectiveness in terms of the lessened pain intensity, measured on a visual analogue scale (VAS), experienced by patients with UCR, at 9 weeks after beginning treatment. As secondary specific goals, we intend to: a) evaluate the effectiveness in terms of lessened pain intensity, measured on the VAS, at 6 months after beginning treatment; b) evaluate the effectiveness in terms of improvement recorded on the Spanish version of the McGill Pain Questionnaire (MPQ-SV), at 9 weeks and at 6 months after beginning treatment; c) evaluate the effectiveness in terms of patient-perceived improvement (PPI) at the end of the treatment; d) evaluate the impact on time lost off work due to sickness; e) evaluate the credibility of the technique and patients' expectations, as well as its relation to the results; f) evaluate the effectiveness in terms of reduced consumption of analgesic and anti-inflammatory medication; g) evaluate the effectiveness in terms of improved health-related quality of life (SF-12); h) compare the effectiveness of the technique with regard to the healthcare professionals' degree of training and practice; i) compare the characteristics of the Lattinen test and the MPQ-SV; j) analyze the cost-effectiveness of treatment with auricular pressure for patients with UCR.

This study is funded by the *Instituto de Salud Carlos III*, *Fondo de Investigaciones Sanitarias *(File No. PI0790058) and by the Health Ministry of the Andalusian Regional Government (File No. 00462007).

## Methods

### Design

Randomized controlled multicentre prospective study, with two parallel arms, to compare real auriculotherapy using vaccaria seeds (in accordance with traditional methods and with individual diagnoses) and placebo auriculotherapy. The patients will be blinded to both treatment methods. Analysis of the results will be carried out by professionals who will be blinded with respect to the allocation of patients to the different treatment groups.

### Duration of Study

May 2008–December 2009

### Subjects

Patients, recruited by the 15 doctors and nurses participating in the study at 10 Primary Healthcare Centers belonging to the Andalusian Public Health System (Sevilla-Sur Healthcare District), with symptoms of uncomplicated chronic rachialgia.

The patients included will be aged at least 18 years, with chronic uncomplicated muscular-skeletal rachialgia (neck, mid-back or lower back), diagnosed by clinical background and physical examination, and who have not previously received treatment with auricular implants. Exclusion criteria will include the protrusion or prolapse of one or more intervertebral discs with concurrent neurological symptoms; infectious spondylopathy; previous surgery of the spinal column; rachialgia caused by inflammatory disease, malign or autoimmmune; congenital deformities of the spinal column, except mild degrees of scoliosis or lordosis; vertebral fractures; spinal stenosis; spondylolysis or spondylolystesis; skin complaints in the auricular pavilion or allergy to sticking plaster; pregnancy; lawsuits brought by reason of rachialgia; incapacity to fill in the questionnaires or respond to the evaluator's questions.

The ethical validity of this study has been analyzed and approved by the Andalusian Government Committee for Clinical Trials, following the approval of the corresponding Research Commission at each of the participating Clinics. The study design takes into account the fundamental principles set out in the Helsinki Declaration, and those of the Council of Europe Convention concerning human rights and biomedicine, as well as the requirements under Spanish law in the field of biomedical research, the protection of personal data, and bioethics. All the patients involved must sign their informed consent to the proposed clinical research procedures. During the course of the study, audits will be performed as required by the relevant Research and Ethics Committee, as well as those of each Clinic's Quality Committee, independently of any external audits (such as that of the research financing body) that may be necessary.

### Randomization and treatment allocation

Sampling will be by consecutive selection in accordance with the inclusion-exclusion criteria, for a period of 12 months until the sample is complete. The randomized allocation to each of the two branches of the study will be carried out using specialized computer software (Epidat v. 3.1), at the Research Unit of the Costa del Sol Hospital (Málaga, Spain), in a 1:1 plan (true auricular pressure: placebo) in blocks of 6, stratified by therapist. Neither the clinics nor the healthcare professional participating in the study will be involved in the randomization process, and the randomization sequence will remain concealed until the end of the study. Patients who meet the criteria for inclusion and who sign their informed consent will be included in the study. After inclusion in the study, the healthcare professional will call the randomization clinic at which the patient is registered, and will be informed, both by phone and by fax, which of the two study branches the patient has been assigned to. This procedure ensures that the randomization process is not influenced by the healthcare personnel participating in the study. The success of the blinding of the patients will be evaluated at the end of the treatment period.

### Sample size

In calculating the sample size, a power of 90% and an α value of 5% were assumed, to detect a mean change in pain intensity, measured on the 100 mm visual analogue scale, of 32.3 mm between the initial and final values among the experimental group, and a mean reduction of 19.8 mm among the control group (standard deviation 31.9 mm), on the basis of the results of a pilot experiment carried out beforehand at the Pain Treatment Unit at the Dos Hermanas Healthcare Clinic between April and December 2006. These assumptions require a sample size of 140 patients per group, in a design with two equal groups. We propose to recruit 400 patients in order to allow for a 30% drop out rate.

### Treatment

The 15 healthcare professionals who will participate in the study (doctors and nurses) have taken a training course in auricular pressure techniques for the treatment of UCR, with a total of 15 hours' theory and 15 hours' practical content. Of these 15 professionals, 5 had also received specific training in acupuncture and related techniques, with an average of 320 hours theoretical training and over 100 hours' clinical practice with these techniques. All the patients will be allowed to use symptomatic medication for pain relief in case of need.

The patients in both groups will be called 8 times for treatment (once a week) for the auricular implants to be inserted.

#### A) True auriculotherapy using pressure with vaccaria seeds (TAP)

Application of auricular implants with vaccaria seeds (vaccaria segetalis Garcke, known in China as Wang bu liu xing) as an individualized form of sensory stimulation, affixed to the auricular pavilion by means of flesh-coloured sticking plaster. Selection of the auricular points will be made in accordance with the pain characteristics and the sensitivity of the auricular zones, examined using a 250 gr pressure detector [[26], 29]. The patients will be requested to squeeze the implant with their finger 10 times, 3 times a day. These implants will remain in place for one week.

#### B) Placebo auriculotherapy (PAP)

The same protocol will be followed, under the same conditions as for TAP, but with the application of sticking plaster over inactive black plastic discs, with a diameter of 1.5 mm, simulating the appearance of the auricular implants used in the TAP.

Any adverse reactions or side effects that may occur will be recorded in the corresponding data logbook, stating details of the reaction, and date of occurrence.

**Figure 1:**
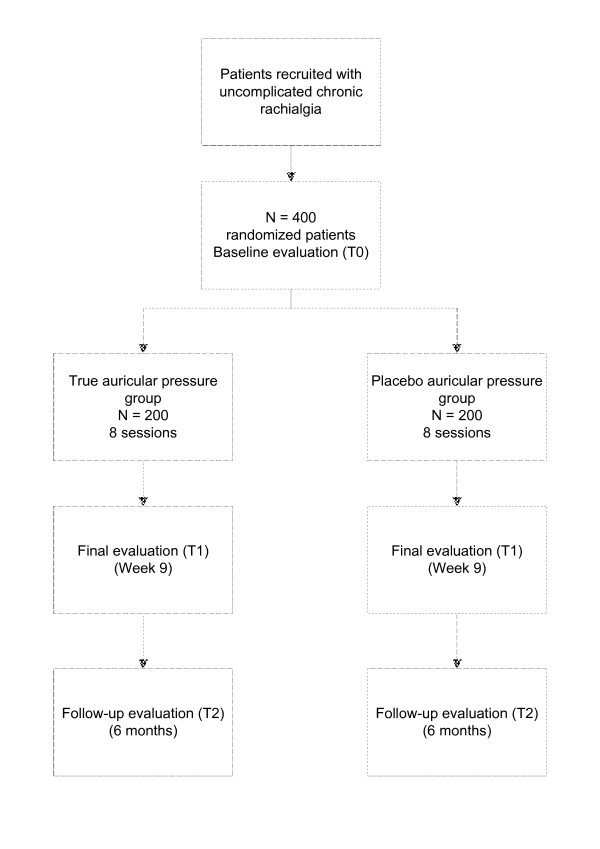
**Flow diagram for the study**. Work scheme with description of assessment visits and times.

The same time should be dedicated to the patients in each of the groups, as should that employed in the pre and post-session evaluations (Fig. 1).

### Results measures

Each patient will fill in a self-response questionnaire before beginning the treatment (T0), at one week after ending treatment (T1) and after six months (T2). This questionnaire will include one-dimensional data (100 mm visual analogue scale) and multidimensional data (McGill Pain Questionnaire, Spanish version [30]) on rachialgia, medication currently being taken and health-related quality of life (SF-12), together with information on days off work due to rachialgia. Additionally, before beginning treatment, the patients will be asked to fill in a form on sociodemographic questions. Any adverse effects of the study techniques, and any use of rescue medication by the patient will also be recorded. During the initial evaluation, an anamnesis will be obtained on the characteristics of the rachialgia, and note will be taken of the duration of the pain. In each treatment session, the therapist will record the five pain-related variables that constitute the Lattinen test (LT), which is widely used in pain treatment clinics in Spain [31]. These variables are: pain intensity, pain frequency, consumption of analgesics, level of activity, and nocturnal hours' sleep. Each item is scored on a Likert scale from 0 to 4; thus, the sum of the five variables may range from 0 to 20 points, with higher scores reflecting greater pain severity. In addition, the auricular points selected for each patient will be recorded (Table 1).

**Table 1:** Data collection schedule. Measurement times and variables used in the study.

Visit No.	T0	Weekly treatment								T1	T2
Week No.	0	1	2	3	4	5	6	7	8	9	24
Data provided by the patient											
Pain intensity (VAS 100 mm)	X									X	X
Multidimensional pain evaluation (MPQ-SV)	X									X	X
Quality of life (SF-12)	X									X	X
Analgesics and antiinflammatory drugs prescribed	X									X	X
Sociodemographic variables	X										
Credibility and expectations				X							
Improvement perceived by the patient										X	
Assessment of blinding effectiveness										X	
Data provided by the therapist											
Multidimensional pain evaluation (LT)	X	X	X	X	X	X	X	X	X	X	X
Auricular points used		X	X	X	X	X	X	X	X	X	
Adverse events		X	X	X	X	X	X	X	X	X	
Economic evaluation											
Direct and indirect costs: healthcare professionals and patients	X									X	X

VAS 100 mm: Visual analogue scale (0–100 mm); MPQ-SV: McGill Pain Questionnaire, Spanish version; LT: Lattinen test

#### Primary outcome measure

Changes in pain intensity, measured on the 100 mm visual analogue scale (VAS) [32], at 9 weeks after beginning treatment.

#### Secondary outcome measures

▪ Changes in pain intensity, measured on the 100 mm VAS, at 6 months after beginning treatment.

▪ Changes in the McGill Pain Questionnaire (MPQ), at the end of treatment and after 6 months. The MPQ pain evaluation index consists of 64 pain descriptors divided into 19 subclasses. Within each subclass, the descriptors are classified by intensity.

▪ Satisfaction on the improvement perceived by the patient (PPI) [33], scored by the patient on a 7-point Likert scale as follows: 1 = extremely satisfied; 2 = very satisfied; 3 = moderately satisfied; 4 = no strong feelings either way; 5 = somewhat unsatisfied; 6 = very unsatisfied; 7 = extremely unsatisfied.

▪ Changes in health-related quality of life, according to the Spanish version of the 12-item Short Form health survey, Version 2, at the end of treatment and after 6 months. This questionnaire is of a generic type, derived from SF-36, which has been validated for use in Spain [34], and which enables the quantification of quality of life in 8 dimensions (physical function, physical role, pain, general health, vitality, social function, emotional role, mental health) and expresses two summary components (physical and mental).

▪ Changes in the results of the Lattinen test and in the consumption of analgesics and NSAIDs (whether or not prescribed by the GP), at the time of randomization, after each treatment session, at the end of treatment and after 6 months.

#### Control of blinding and assessment of treatment credibility

The patients will be asked to answer the following questions:

• Expectations and confidence in the treatment (ECT), evaluated after the third auricular pressure session, and scored on the original scale of Borkovec and Nau [35] with four items, scored on a numerical scale of 0 to 10 (0: totally disagree; 10: totally agree): (1) Are you confident that this treatment will relieve the pain you feel?; (2) Does the treatment seem to be a logical one?; (3) Would you recommend this treatment to a friend or relative suffering the same complaint?; (4) Do you believe this treatment would be a possible option for dealing with other problems?

• Verification of the patient's blinding (VB) [36]. After the final treatment session, the patient will be asked: "What treatment do you think you were given?" The possible answers are: 1 = true auricular pressure; 2 = sham auricular pressure; 3 = Not sure.

#### Sociodemographic variables

The following data on sociodemographic variables will be recorded: age, race, sex, marital status, educational level, financial level and occupational activity.

### Data collection and analysis

#### Data collection

The data will be compiled in a general questionnaire addressing each of the study variables, both in the self-administered formats and in those applied by direct observation. These data will be sent to the study coordinator at the end of the follow-up evaluation of each patient.

#### Recording study data and information on adverse effects

A record will be kept of any side effects experienced, and of possible adverse events arising from either the experimental or the pharmacological treatment.

#### Statistical analysis

A comparison will be made of the baseline variables for each of the groups, to test the homogeneity obtained from the random allocation to groups, in terms of differences of the means and of proportions. The magnitude of the difference in the possible imbalance produced by the random allocation between the two groups will be quantified in terms of ratios of the means and of proportions (using that of the placebo group as a reference level), and the final adjustment will be made by secondary analyses with multiple linear regression models.

In the unadjusted analysis, significance tests will be performed for comparison among k samples (parametric or otherwise, depending on whether or not the distribution of the outcome variables is asymmetric, and on the homogeneity of their variances), taking that of the placebo group as a reference level, and using comparison tests for differences of the means in the primary outcome measure (pain intensity on the 100 mm VAS), both for the inter-group comparisons (for independent samples) and for comparisons between the initial and final levels in each group (in this case, using tests for non-independent or paired samples).

Linear regression models will be constructed for the primary outcome measure, adjusted by the baseline level and using ITT analysis. The group variables will be included, taking that of the placebo group as a reference level, together with the results recorded by the medical professionals, and the sociodemographic data (age and sex), and the baseline levels for the variables related to the severity of the process (pain intensity and frequency). Adjustments will be made for possible confounders, using criteria of statistical significance and of confusion. The detection of possible interactions with the treatment group variable will be evaluated using criteria of statistical significance for the corresponding interaction terms. The level of statistical significance will be set at α < 0.05. The model will be reconstructed, removing the observations with Cook's distances exceeding the 90^th ^percentile of the distribution, in order to test the consistency of the results.

An economic analysis will also be carried out, from the standpoint of the interests of the Andalusian Public Health System.

## Discussion

This is an experimental study in which treatment with auricular implants is compared with that given to a placebo group, set up in such a way that the patient has the sensation of being given the real treatment. This control group, due to the fact of receiving specific attention and due to the effects of sensorial stimulation (the pressure on the implants) may have some positive results. This circumstance is contrary to the study's initial hypothesis, but to some extent reflects the inevitable placebo effect.

In order to obtain a sufficient number of patients, the study is being carried out at various healthcare clinics. It will be necessary to perform a stratification function so that all the clinics will receive equal proportions of patients in each of the two treatment branches. The patients will be asked not to obtain any kind of alternative treatment, but they will be allowed to continue taking any analgesic or anti-inflammatory medication needed, depending on the pain intensity experienced.

An important limitation that may affect this study is that of non-compliance with the prescribed treatment; patients might not attend a treatment session for any of several possible reasons. The principle outcome measure will be based on ITT, but a per protocol analysis will also be performed.

The sample size has been calculated on the assumption of a 30% drop out rate, but it will be necessary to ascertain that no differential losses occur between the two treatment branches.

## Competing interests

The authors declare that they have no competing interests.

## Authors' contributions

JV conceived the study, designed the study protocol, sought funding and ethical approval and wrote the manuscript. All authors contributed to the research design, read, made critical revisions and approved the final manuscript.

## Pre-publication history

The pre-publication history for this paper can be accessed here:


